# Chronic Clomipramine Treatment Reverses Core Symptom of Depression in Subordinate Tree Shrews

**DOI:** 10.1371/journal.pone.0080980

**Published:** 2013-12-02

**Authors:** Jing Wang, Anping Chai, Qixin Zhou, Longbao Lv, Liping Wang, Yuexiong Yang, Lin Xu

**Affiliations:** 1 Key Laboratory of Animal Models and Human Disease Mechanisms of Chinese Academy of Science & Yunnan Province, and Laboratory of Learning and Memory, Kunming Institute of Zoology, Chinese Academy of Sciences, Kunming, Yunnan, China; 2 KIZ/CUHK Joint Laboratory of Bioresources and Molecular Research in Common Diseases, Kunming Institute of Zoology, Chinese Academy of Sciences, Kunming, Yunnan, China; 3 Kunming Primate Research Center of Chinese Academy of Sciences, Kunming Institute of Zoology, Chinese Academy of Sciences, Kunming, Yunnan, China; 4 Kunming College of Life Science, University of Chinese Academy of Science, Beijing, China; Kunming Institute of Zoology, Chinese Academy of Sciences, China

## Abstract

Chronic stress is the major cause of clinical depression. The behavioral signs of depression, including anhedonia, learning and memory deficits, and sleep disruption, result from the damaging effects of stress hormones on specific neural pathways. The Chinese tree shrew (*Tupaia belangeri chinensis*) is an aggressive non-human primate with a hierarchical social structure that has become a well-established model of the behavioral, endocrine, and neurobiological changes associated with stress-induced depression. The tricyclic antidepressant clomipramine treats many of the core symptoms of depression in humans. To further test the validity of the tree shrew model of depression, we examined the effects of clomipramine on depression-like behaviors and physiological stress responses induced by social defeat in subordinate tree shrews. Social defeat led to weight loss, anhedonia (as measured by sucrose preference), unstable fluctuations in locomotor activity, sustained urinary cortisol elevation, irregular cortisol rhythms, and deficient hippocampal long-term potentiation (LTP). Clomipramine ameliorated anhedonia and irregular locomotor activity, and partially rescued the irregular cortisol rhythm. In contrast, weight loss increased, cortisol levels were even higher, and in vitro LTP was still impaired in the clomipramine treatment group. These results demonstrate the unique advantage of the tree shrew social defeat model of depression.

## Introduction

Major depressive disorder is characterized by low mood, loss of interest or pleasure in normally enjoyable activities, feelings of guilt, and chronic lack of energy. It is also a neuropsychiatric syndrome characterized by impaired structural and synaptic plasticity as well as neuronal damage [1]. According to ICD-10 and DSM-IV diagnostic criteria, a clinical diagnosis requires expression of at least two of the three core symptoms (persistent sadness, loss of interest, fatigue) for at least 2 weeks [2].

Disruption of neuroendocrine function may underlie many symptoms of depression [3]. The hypothalamic-pituitary-adrenal (HPA) axis is overactive in depressed patients, as reflected by elevated plasma cortisol and adrenal gland hypertrophy [4]. The hippocampus provides negative feedback to the HPA axis and is critical for certain forms of learning and memory [5]. Patients with HPA axis dysfunction also exhibit hippocampal atrophy, which may further disinhibit the HPA axis and cause further limbic system dysfunction [6],[7]. Activity-dependent synaptic plasticity is believed to be a neurocellular mechanism underlying some forms of learning and memory, and hippocampal synapses demonstrate several forms of synaptic plasticity [8],[9]. In experimental animals, stress impairs learning and memory and alters the threshold or duration of synaptic plasticity. For example, stress impairs long-term potentiation (LTP) and enhances long-term depression (LTD) in the hippocampus in vitro and in vivo [10]–[12].

Rodents and non-human primates are used extensively as models to study the behavioral, neuroendocrine, and neurological changes associated with depression. Animals exposed to chronic stress exhibit behavioral endophenotypes of depression and physiological changes similar to those observed in human patients. Moreover, these anomalies are responsive to antidepressant treatment. Stressful life events are a major cause of depression [13]–[15]. Early life stress can trigger lasting depression-like behaviors in non-human primates [16]. While higher primates may be the most robust model of human psychiatric disease, prohibitive cost, the long experiment cycle, and animal rights issues have led to the establishment of rodent models that exhibit both similar physiological responses to stress and behavioral phenotypes useful for studying the physiological basis of depression and the efficacy of antidepressant drugs. Rodent models can be divided into three categories: models of acute stress, models of secondary or iatrogenic depression, and chronic stress models [17]. The forced swim test and tail suspension test are used mainly to test antidepressant action, but they do not fully cover the complexities of human depressive symptoms. In contrast, the unpredictable chronic mild stress (UCMS) model shows predictive and construct validity for depression [18],[19]. However, the neurological, endocrine, and behavioral changes may vary with the UCMS model employed, making analysis of pathogenesis difficult. Moreover, UCMS stimuli (electric shocks, restraint, etc.) have little resemblance to stressful life events in the natural environment. Almost all studies of UCMS models have focused on the 4 to 5 weeks after mild stress treatment [20]. Thus, a new animal model with a single behaviorally relevant stressor and a clinically relevant time scale is required.

Phylogenetic analysis of 15 mammalian species, including six primates, has shown that the tree shrew is the closest relative of primates [21],[22], and several recent reports indicate that chronic psychosocial stress in tree shrews is a robust model of clinical depression. Chronic psychosocial stress can cause body weight loss, elevated cortisol, adrenal gland hypertrophy, reduced testosterone [23],[24], hippocampal atrophy, and downregulation of glucocorticoid and mineralocorticoid receptors in these animals, as well as depression-like behavioral changes [25]–[27]. However, the core symptoms of depression such as anhedonia, and synaptic plasticity of subordinate tree shrews in this model has not been studied. Here, we used the chronic social defeat model in male Chinese tree shrews (*Tupaia belangeri chinensis*) to investigate whether chronic antidepressant treatment improves core symptoms of depression, synaptic plasticity and other depression-like behaviors. To better mimic the time scale of human depression, we measured activity during 4 weeks of clomipramine administration and after a 1-week recovery (no treatment) period.

## Materials And Methods

### Animals And Ethics Statement

Adult male Chinese tree shrews (*Tupaia belangeri chinensis*, N = 18) weighing 130−160 g were obtained from a breeding colony at the Animal House Center of the Kunming Institute of Zoology. All animals were provided ad libitum access to food and water. They were housed individually in thermoregulated rooms (T: 25∼27 °C, RH: 55%∼70%) under a 12 h light/dark cycle (light, 8:00–20:00; dark, 20:00−8:00). All animal care and experimental protocols were approved by the Animal Care and Use Committee of Kunming Institute of Zoology, Chinese Academy of Sciences, P. R. China.

### Experimental Procedures

**Figure 1:**
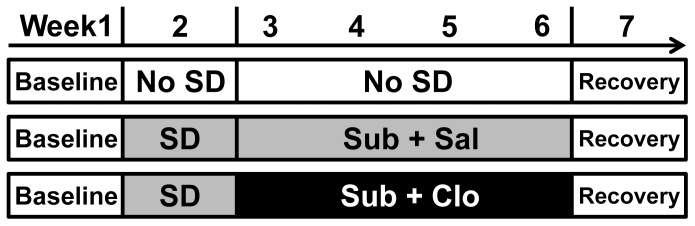
The experimental design. The experiment included three experimental groups, Naïve, Subordinate+Saline (Sub+Sal), and Subordinate+Clomipramine (Sub+Clo), and four phases. Phase 1 consisted of a 7 day stress-free period during which the animals adapted to the experimental environment. Phase 2 was the social defeat (SD) phase during which animal pairs in the Sub+Sal and Sub+Clo groups housed separately in connected cages were allowed direct access to fight for social dominance. In contrast, Naïve group tree shrews remained undisturbed. Phase 3 was the drug administration plus SD phase lasting 28 days during which the subordinate (Sub) tree shrews were exposed to daily social defeat stress as before but also treated daily with oral clomipramine (50 mg/kg/day) or saline (1 ml/kg/day). The final recovery phase consisted of a 7 day period with neither social defeat nor drug treatment.

The experimental design was similar to that described previously [24],[28],[29]. In brief, the experiment included four phases (Fig. 1): baseline (Week 1), chronic social defeat (SD, Week 2), drug or vehicle administration with social defeat (Weeks 3−6), and recovery (Week 7). Animals were divided into three groups of six: Naïve, Subordinate+Saline (Sub+Sal), and Subordinate+Clomipramine (Sub+Clo). For the first week (baseline), tree shrews were adapted to a paired cage (100 cm×68 cm×86 cm, w×d×h) consisting of two individual cages connected by a door normally blocked by a wire mesh partition. The front of the paired cage was made of glass to allow observation of animal behavior. During the SD phase, animals in the Sub+Sal and Sub+Clo groups were allowed daily direct access for 1 h at an unpredictable time between 9:00 and 18:00 by removing the barrier. This resulted in a brief fighting episode to establish the social hierarchy. The avoider tree shrew was regarded as the subordinate of the pair and the other as the dominant. For the remainder of each day, both animals were exposed to visual, auditory, and olfactory cues. After seven days of daily social defeat, subordinate tree shrews were treated with clomipramine (50 mg/kg per day, Sigma) or vehicle (0.9% saline) orally for the next 4 weeks (drug phase) while still experiencing daily social defeat. Drug or vehicle was administered between 07:45 and 08:00 A.M. [19].

Animals of the Naïve group were individually housed and left undisturbed in the same type of cage and under the same environmental conditions (T, RH) as the two experimental groups. Every weekend, animals were weighed, morning urine samples collected, sucrose solution and water consumption measured, and behavior videotaped.

### Measurements

**Body weight and behavioral analysis.** Tree shrews were weighed in the morning and videotaped between 17:30−18:00 P.M. through the glass side of the paired cage. A Noldus EthoVision XT Version 8.0 video tracking system (Wageningen, the Netherlands) was set to analyze locomotor behaviors, which was used to assess motor fatigue and/or agitation [30],[31], and self-grooming behavior. Locomotion number was recorded, including the number of jumping in the activity area and the number of passages between the sleeping-box and activity area of the cage. This recording schedule was chosen to avoid the confounding effects of human activity around the cages by staff during weekdays.

**Sucrose consumption test.** In our previous study, we found that 5% was the best sucrose concentration for the tree shrew sucrose consumption test [32]. All experimental animals were adapted to 2% sucrose solution 24 h before the test, which was performed once a week. During the sucrose consumption test, animals were given two bottles, one containing 200 ml of 5% sucrose solution and the other 200 ml of water. The bottle position was changed randomly (left vs. right). The volumes of sucrose solution and water consumed over the next 24 h were recorded. Sucrose preference was calculated as follows:

**Analysis of urinary cortisol**. Urine samples were collected between 7:45−08:00 A.M. once every weekend and 12 h urine samples were collected from 08:00 to 20:00 on the final week. Urine samples were stored at –20 °C until analysis and free cortisol measured by an Iodine [^125^I] cortisol radioimmunoassay kit (Beijing North Institute of Biological Technology, China) on a γ radioimmunity counter (GC-2010, Zonkia, Anhui, China).

### Electrophysiological Recording

**Slice preparation.** After the recovery week (Week 7), hippocampal slices were prepared from control (Naïve), clomipramine-treated (Sub+Clo), and saline-treated (Sub+Sal) tree shrews using procedures described previously [33],[34]. Briefly, the animal was deeply anesthetized with diethyl ether. After decapitation, the brain was carefully removed. Coronal hippocampal slices were prepared at 350 µm using a vibratome (Leica, VT 1000S) in ice-cold oxygenated (95% O_2_/5% CO_2_) cutting medium containing (in mM) 206 sucrose, 2.5 KCl, 1.25 NaH_2_PO_4_, 26 NaHCO_3_, 10 D-glucose, 3 MgSO_4_, 1 ascorbic acid, and 2 CaCl_2_ H_2_O (pH 7.2−7.4, 300−310 mOsm). After submerged incubation for 45 min at 31 °C in cutting solution, slices were transferred and submerged in a holding chamber containing oxygenated (95% O_2_/5% CO_2_) ACSF (in mM: 120 NaCl, 2.5 KCl, 1.25 NaH_2_PO_4_, 26 NaHCO_3_, 10 D-glucose, 2 MgSO_4_, 1 ascorbic acid, and 2 CaCl_2_•H_2_O, pH 7.2−7.4, 300−310 mOsm) and incubated at room temperature (RT) for at least 30 min before recording.

**Field excitatory postsynaptic potential recording and data analysis.** Field excitatory postsynaptic potentials (fEPSPs) in the hippocampal CA1 area were recorded in a chamber maintained at RT and superfused with standard ACSF plus 100 µM picrotoxin (Sigma) as described in our previous study [35]. Evoked fEPSPs of about 50% of maximum amplitude were recorded from the stratum radiatum in response to 0.1 ms stimuli from an electrode made from a pair of twisted Teflon-coated 90% platinum/10% iridium wires (0.025 mm diameter, World Precision Instruments) placed in the Schaffer collateral/commissural (SC) pathway. Only slices with a maximum fEPSP amplitude greater than 0.5 mV were included in this study. Signals were amplified using a Multiclamp 700B amplifier (Axon CNS Molecular Devices), low-pass filtered at 2 kHz, and digitized at 10 kHz. Recording electrodes (resistance, 1–3 MΩ) were pulled from borosilicate glass capillary tubes (1.5 mm outer diameter, 0.84 mm inner diameter, World Precision Instruments) using a Brown-Flaming micropipette puller (P-97; Sutter Instruments Company) and filled with standard ACSF. A stable 20 min baseline was established at 0.033 Hz and LTP induced by high frequency stimulation (HFS, three trains of 1-s stimulation at 100 Hz with 20 s inter-train intervals) at the same stimulation intensity. The magnitude of LTP was calculated from the average of the last 10 min of recording (20 individual sweeps 50−60 min post tetanus) and reported as the (%) mean ± SEM of baseline fEPSP amplitude.

### Statistical Analysis

Data was analyzed using SPSS 19.0 (SPSS, Inc., Chicago, IL, USA). Differences in LTP amplitude between Sub+Sal and Sub+Clo groups were tested for statistical significance by one-way ANOVA followed by post hoc Fisher’s LSD test. Each physiological or behavioral parameter was measured once weekly, averaged within groups (Naïve, Sub+Sal, and Sub+Clo), and expressed as mean ± SEM. To judge the success of the depression model, the effects of 1 week of social defeat on the various parameters and behaviors measured were tested by one-way ANOVA followed by post hoc Fisher’s LSD test. To assess the therapeutic efficacy of clomipramine, we analyzed data from Weeks 2−6 using both a repeated measures ANOVA followed by within-group analysis to investigate the possible interaction of experimental group and time (group × time) and a one-way ANOVA followed by Fisher’s LSD test to compare group means. Data from the last 2 weeks, including the last week of treatment (saline or clomipramine) and the recovery week, were compared by two-way ANOVA to assess the efficacy of clomipramine during the recovery phase. The significance level for all tests was set at *P*<0.05.

## Results

### Clomipramine Did Not Rescue Decreased Body Weight Associated With Social Defeat

**Figure 2:**
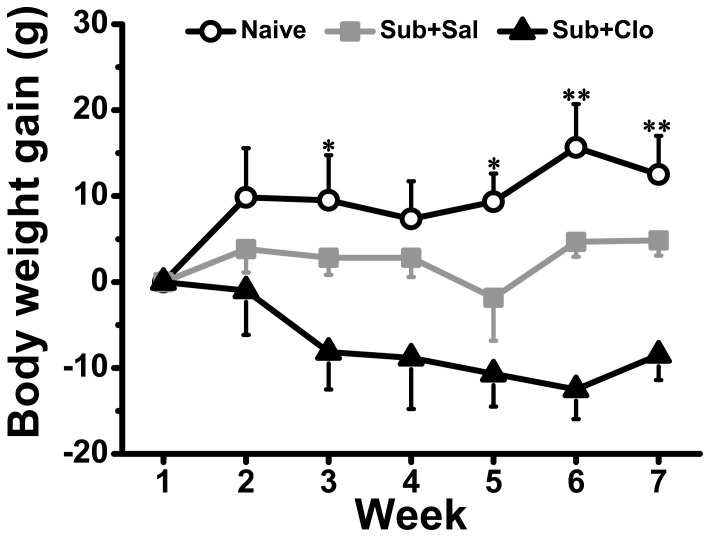
Clomipramine did not reverse weight loss associated with chronic social defeat stress. In the Naïve group, body weight increased over time. Chronic social defeat inhibited weight gain and this effect was not altered by clomipramine. After 4 weeks of clomipramine administration, body weight was still significantly lower in subordinate tree shrews. *p<0.05, **p<0.01.

Chronic social defeat caused a modest but statistically significant decrease in body weight gain (Fig. 2), and clomipramine treatment did not reverse this effect [group: F_(2,15)_ = 7.264, *P* = 0.006; time: F_(4,60)_ = 1.660, *P* = 0.171]. Subordinate animals were still significantly lighter than naïve subjects even after 4 weeks of oral clomipramine treatment (Week 6) [F_(2,15)_ = 14.937, *P* = 0.000] and reduced body weight was maintained in both Sub+Sal and Sub+Clo groups after the 1-week recovery period (Week 7) [F_(2,30)_ = 25.502, *P* = 0.000].

### Clomipramine Partially Reversed Anhedonia And Fatigue Associated With Chronic Social Defeat

**Figure 3:**
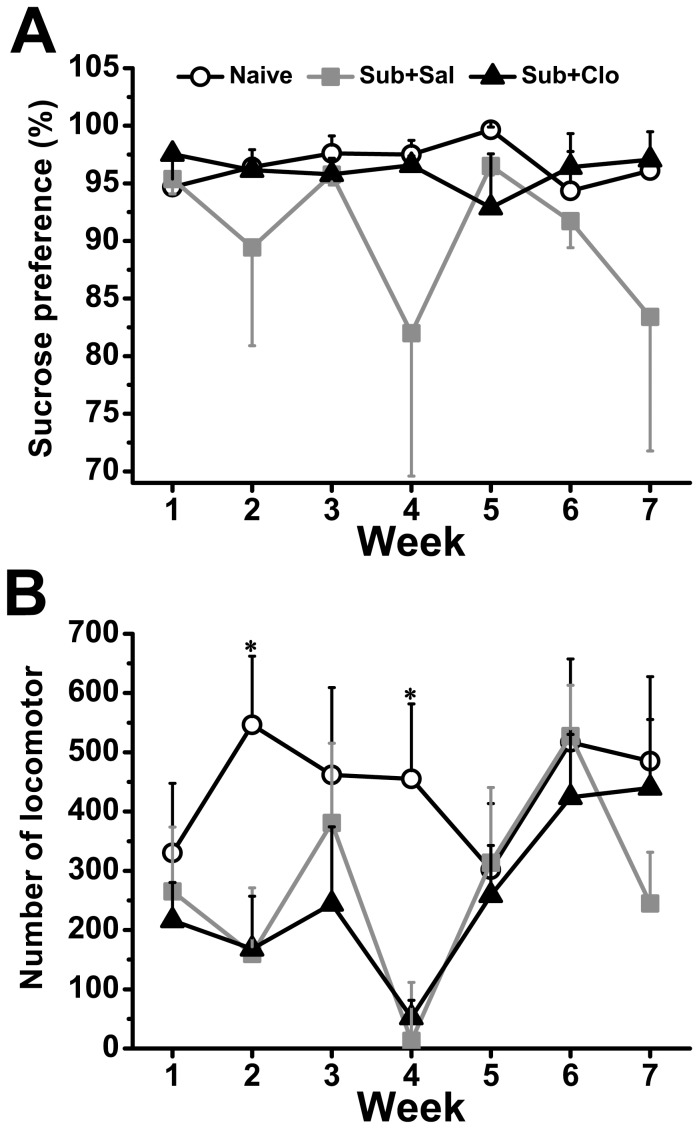
Clomipramine ameliorated anhedonia and psychomotor instability associated with chronic social defeat stress. (A) All groups preferred 5% sucrose to water, but preference was significantly higher in the Naïve group compared to the Sub+Sal group. Normal sucrose preference was restored in the Sub+Clo group. (B) In the unstressed Naïve Group, locomotor activities were stable. Stress induced an unstable fluctuation in locomotor activity in both Sub+Sal and Sub+Clo groups, but the Sub+Clo group exhibited locomotor activity levels similar to the Naïve group by the recovery phase. *p<0.05.

To examine whether clomipramine can ameliorate depression-like behaviors in tree shrews, we compared sucrose preference (a measure of anhedonia) and locomotion (to assess motor fatigue and/or agitation) (Fig. 3). Anhedonia was measured by reduced preference for 5% sucrose over water. While sucrose was preferred by all three groups as evidenced by the ratio of 5% sucrose to water consumed (≈80%−95% of total fluid consumed was the sucrose solution), the Sub+Sal group exhibited reduced sucrose consumption compared to Naïve animals. Comparison among groups showed no significant difference in 5% sucrose consumption [F_(2,15)_ = 2.454, *P* = 0.120; Fig. 3A]. Post hoc analysis revealed a clear decrease in sucrose preference in the Sub+Sal group compared to the Naïve group (*P* = 0.050) but no difference between the Sub+Clo and Naïve groups (*P* = 0.596), as well as Sub+Sal group (*P* = 0.133), indicating that clomipramine reversed anhedonia in this animal model.

Fatigue was reflected by reduced locomotor activity, including less jumping and fewer times passing from the sleeping-box to the activity area. One week of social defeat caused a significant reduction in locomotion number [F_(2,15)_ = 4.331, *P* = 0.033; Fig. 3B]. During the next 4 weeks of daily fighting and treatment, the Naïve group showed a relatively steady locomotor activities, but neither the Sub+Sal nor Sub+Clo groups exhibited unstable fluctuations in locomotion [time × group: F_(8,60)_ = 2.159, *P* = 0.044; Fig. 3B]. Locomotion decreased after one week stress exposure and then rose at the next week. At the 4^th^ week, the locomotion decreased again, and higher in the next two weeks. In the recovery phase, the Sub+Sal group exhibited lower locomotor activity than the Naïve group, while activity in the Sub+Clo group recovered to Naïve group levels. Thus, chronic social defeat caused anhedonia and motor fatigue, and these depression-like behaviors were ameliorated by clomipramine.

### Clomipramine Had No Effect On Urinary Cortisol And Self-Grooming Behavior

**Figure 4:**
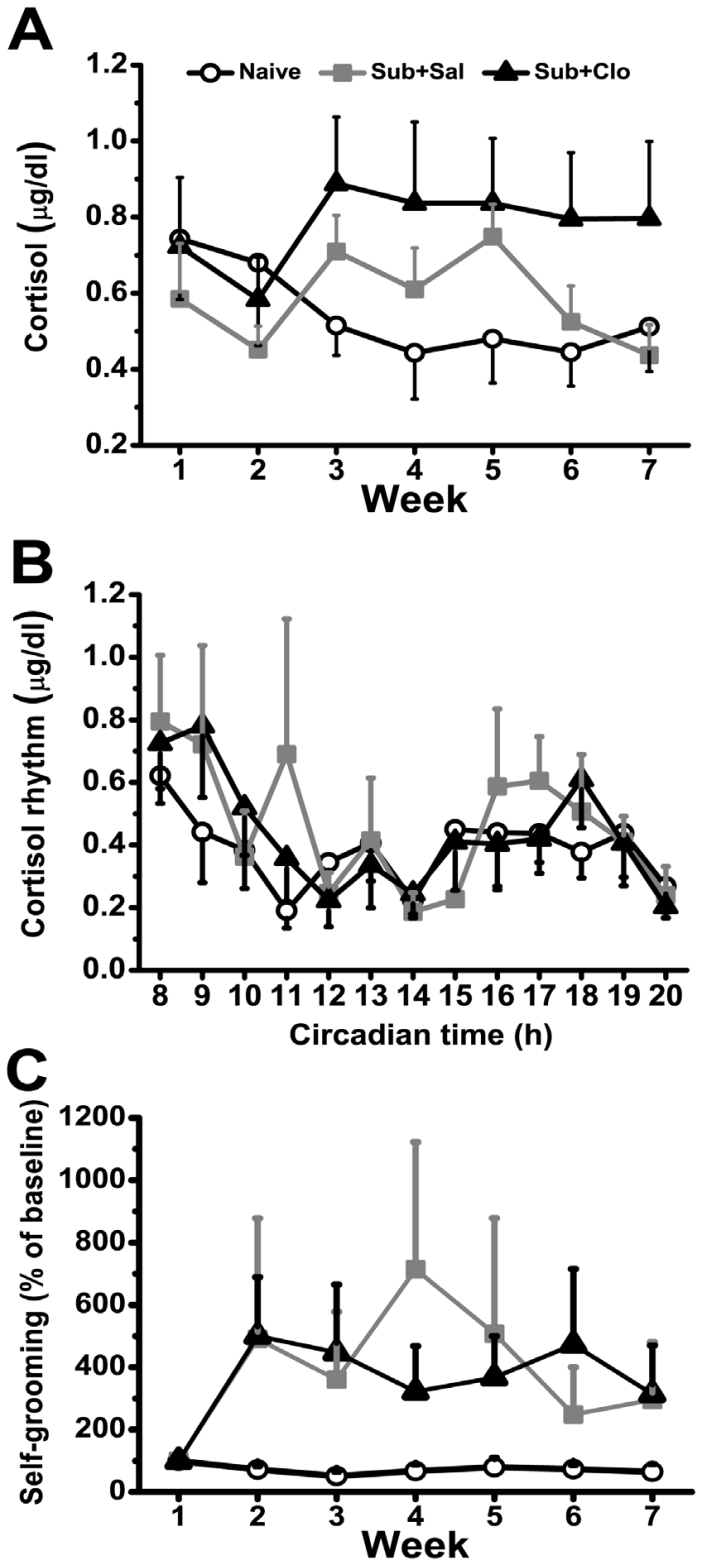
Effect of chronic social defeat and clomipramine on urinary cortisol and self-grooming behavior. (A) In the Naïve group, the concentration of urinary cortisol remained constant throughout the entire experiment. In the Sub+Sal group, stress induced a sustained elevation in urinary cortisol. Similarly, urinary free cortisol was elevated throughout the whole stress period in the Sub+Clo group. (B) On Week 7, urinary cortisol rhythm was measured in all groups. Clomipramine reduced urinary cortisol at 16:00–17:00, the time of peak activity for tree shrews. (C) Self-grooming behavior of both Sub+Sal and Sub+Clo groups were increased after 1 week social defeat. From 2 to 6 weeks, the number of autogrooming behavior in Sub+Sal group animals showed fluctuated relative to Sub+Clo group.

Chronic social defeat resulted in elevated urinary cortisol (Fig. 4A). Urinary cortisol recovered over Weeks 6 to 7 to near control levels in the Sub+Sal group but remained elevated in the Sub+Clo group. Thus, clomipramine did not reverse this sign of stress-induced HPA dysregulation, and may have even prolonged it. From Week 2 to Week 6, the comparison among groups revealed no significant difference in morning urinary cortisol levels [F_(2,15)_ = 2.948, *P* = 0.083; Fig. 4A]. However, post hoc analysis revealed significantly higher urinary cortisol in the Sub+Clo group after 4 weeks of clomipramine treatment compared to the Naïve group (*P* = 0.030). Whereas, there had no significant different between Sub+Sal and Naïve group (*P* = 0.407), as well as Sub+Clo (*P* = 0.144). The high level of urinary cortisol had lasted through the recovery phase [F_(2,30)_ = 3.690, *P* = 0.037].

To investigate whether the cortisol level of vehicle-treated subordinate tree shrew were return back to normal, we tested the cortisol rhythm of all animals at the recovery phase (Fig. 4B). The cortisol rhythm of Sub+Sal group showed disorder comparing with Naïve group, which was partially normalized by clomipramine, especially at the time of peak activity (16:00 to 17:00).

Self-grooming behavior, as a behavioral feature often related to HPA axis activity, was also analyzed (Fig. 4C). Compared with Naïve group, the autogrooming of Sub+Sal group was fluctuated. After 28 days clomipramine treatment, it was tend to stable.

### Clomipramine Did Not Rescue Impaired Hippocampal Ltp In Subordinate Tree Shrews

**Figure 5:**
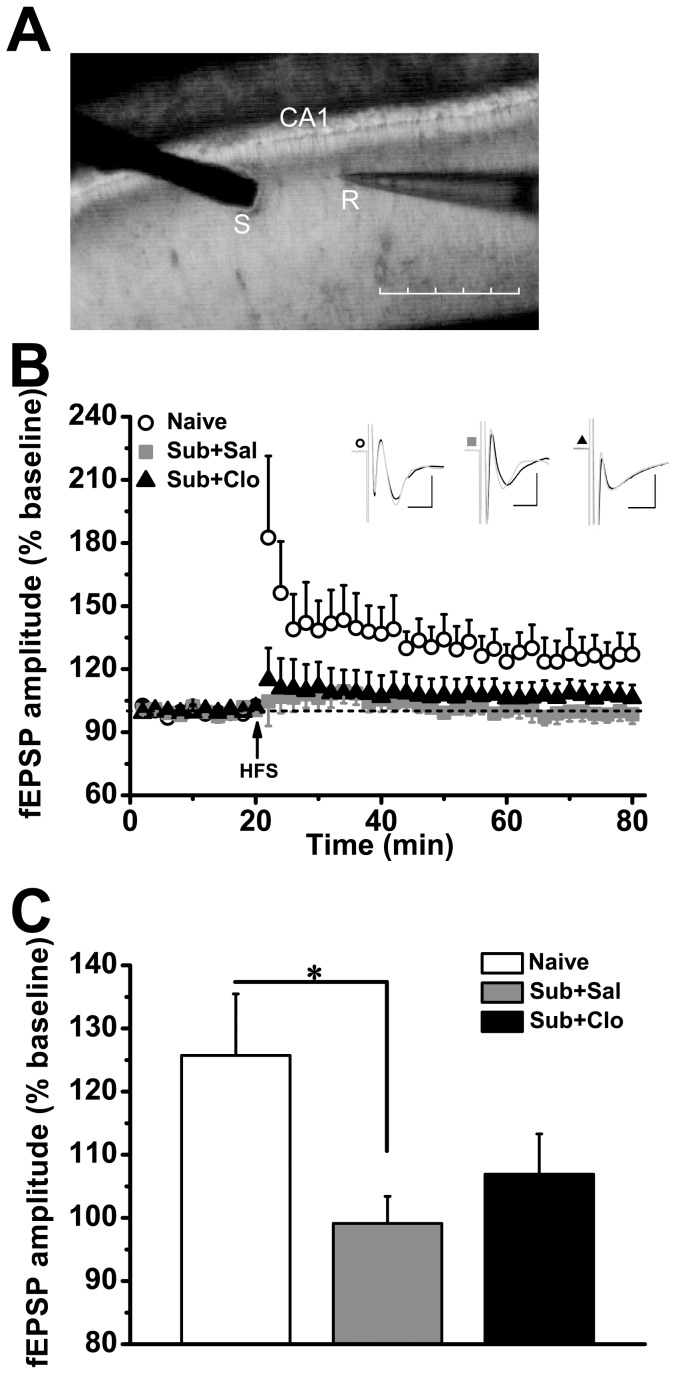
Clomipramine did not rescue deficient SC-CA1 long-term potentiation in subordinate tree shrews. (A) Micrograph showing the positions of the stimulating electrode (S) and the recording electrode (R) in the tree shrew hippocampal CA1 (scale bar: 350 µm). (B) LTP induced by high-frequency stimulation (HFS) in Naïve (N = 4 animals, n = 6 slices), Sub+Sal (N = 4, n = 5), and Sub+Clo tree shrews (N = 4, n = 7). Inset above: Typical fEPSPs recorded at baseline and 50−60 min post-HFS (horizontal bar: 5 ms, vertical bar: 0.5 mV for each group). (C) There was no significant difference in LTP at 50−60 min post-HFS between the Sub+Sal and Sub+Clo group. Both groups exhibited impaired LTP compared to the Naïve group. *p<0.05 compared to the Naïve group.

Activity-dependent changes in synaptic strength, including LTP and LTD, can be altered by stress. As expected, chronic social defeat impaired LTP [F_(2,15)_ = 2.987, *P* = 0.081; Fig. 5C]. Post hoc analysis revealed that 5 weeks of social defeat stress impaired in vitro LTP in the hippocampal SC−CA1 pathway of the Sub+Sal group (*P* = 0.035) and Sub+Clo group (*P* = 0.087) compared to the Naïve group, and LTP in the Sub+Clo group was not significantly different from the Sub+Sal group (*P* = 0.525).

## Discussion

The present study examined whether 4 weeks of daily antidepressant treatment reduced the core symptoms of depression and rescued hippocampal plasticity in the *T. b. chinensis* chronic social defeat model of depression. Chronic social defeat in male tree shrews caused statistically significant decreases in body weight and sucrose preference, unstable fluctuations in locomotor activity and self-grooming behavior, and elevated urinary cortisol. After 4 weeks of clomipramine administration, anhedonia was ameliorated and fluctuations in locomotion and autogrooming behavior normalized. In contrast, irregular cortisol rhythm was only partly restored, weight loss was actually larger, and urinary cortisol higher in the clomipramine treatment group. In addition, clomipramine did not restore the hippocampal LTP deficit associated with chronic social defeat stress. These results highlight the utility of social defeat stress in tree shrews to model depression. We suggest that anti-depressants may ameliorate some depression-like behaviors but not others by selective effects on the neurobiological mechanisms controlling these behaviors. Thus, clomipramine may reduce anhedonia by restoring proper function of the dopaminergic reward pathways but may not rescue forms of learning and memory dependent on hippocampal LTP.

### Clomipramine Treated Two Core Symptoms Of Depression Shared By Humans And Animal Models

Anhedonia, one of the core symptoms of depression, has been widely used in rodent depression model to evaluation degree of depression and effect of antidepressant drug [36]. Reduced preference for sucrose solution over water is a widely used animal model of anhedonia. Anhedonia in both experimental models and depressed patients implies a defective reward system [37] that can be effectively treated by the tricyclic antidepressants (TCAs) [38],[39]. The major difference between tree shrews and rodents is the concentration of sucrose needed to demonstrate stress-induced anhedonia. Most rodent models use 1% sucrose [40], while our previous studies found that tree shrews preferred 5% sucrose [32]. In most models, including the tree shrew chronic social defeat model, stressed subjects exhibit reduced sucrose uptake that is restored by clomipramine or other antidepressants.

Depressed or irritable mood and psychomotor retardation are also common symptoms of depression [41]. Psychomotor agitation is relatively more common in bipolar depression but is also seen in 19% of cases with unipolar depression [42]. The olfactory bulbectomized rodent is thought to be the best model of agitated depression and TCA drugs can partially reverse this symptom, but this is not a stress-related depression animal model [43]–[45]. Previous studies on tree shrews did not report this phenomenon; rather, chronic psychosocial stress always caused a reduction in locomotor activity. Clomipramine did not counteract this effect of stress after 3 weeks of administration, but did after 30 days [46]–[48]. In our study, the Noldus EthoVision XT Version video tracking system revealed a significant decrease in locomotor activity in subordinate tree shrews beginning during the first week of social defeat. As fighting continued, however, we observed unstable fluctuations in locomotor activity in both vehicle- and clomipramine-treated animals compared with relatively steady locomotion in naïve subjects. The unstable fluctuation possibly reflected alternating periods of psychomotor depression and agitation. During the recovery phase, the drug-treated group showed the same level of locomotion as the Naïve group, suggesting that the efficacy of clomipramine was delayed by several weeks, consistent with clinical studies [41],[49] and underscoring the predictive validity of this model of depression.

### Irregular Urine Cortisol And Self-Grooming Behavior Were Stabled By Chronic Clomipramine Treatment

The HPA axis is an essential component of the stress response, but excessive and chronic activation of the axis has been implicated in depression. Hyperactivity of the HPA axis is observed in the majority of patients with depression [50],[51] and can be normalized by administration of clomipramine [52]. Like humans, cortisol is also the main stress-related hormone in tree shrews [32]. In the previous studies, psychosocial stress induced a sustained and significant activation of the HPA axis in subordinate tree shrews, which was decreased by daily treatment of clomipramine [47],[53]. The same as our results, later researches reported chronic social defeat induced a sustained urinary cortisol elevation in northern tree shrews (*T. belangeri*) that was not rescued by clomipramine treatment [48]. Other behavior task such as self-grooming behavior, which is often related to HPA axis activity, is presumably an essential behavior for mammals. Here, we also analyzed self-grooming behavior of tree shrews. The result showed that social defeat stress seemed to increase the self-grooming in Sub+Sal and Sub+Clo groups. Consistent with the unstable locomotion and irregular cortisol level, the self-grooming behavior fluctuated in Sub+Sal group which was stable with clomipramine administration. However, it is different from previous studies by Fuchs et al. [53]. They found that self-grooming of subordinate tree shrews was decreased. The explanation may be the difference between species, individuals, and situations. In the present study, the animal (*T. b. chinensis*) we used was one of subspecies in *Tupaia belangeri,* belonging to genus *Tupaia*, family *Tupaiidae* in the order Scandentia. The results showed that clomipramine administration could stabilize cortisol level, irregular rhythm and self-grooming behavior. It indicated that dysregulation of HPA was eased by chronic clomipramine treatment, suggesting compromised habituation of the HPA axis by clomipramine. Additional studies are required to unravel the pharmacological mechanisms for this effect. Regardless of the mechanism, there was a clear disconnect between some stress-associated responses and others, and this may reflect the specific stress paradigm used. In this model, animals fought daily, which may lead to stress even in the dominant male.

### Chronic Social Defeat Suppressed Synaptic Plasticity In The Hippocampus

Human brain imaging and autopsy studies on the brains of depressed patients have shown marked alterations in the size, cytoarchitecture, and biochemistry of several brain areas involved in the stress response, including regions of the hippocampus, amygdala, thalamus, prefrontal cortex, cingulate cortex, and striatum [41],[54]. Reduced hippocampal volume, loss of excitatory synapses, and dendritic atrophy may explain many of the cognitive deficits in major depression [55]–[57]. Indeed, hippocampus-dependent memory impairment was correlated with hippocampal volume reduction [58]. Chronic psychosocial stress can lead to a reduction in hippocampal volume and downregulation of glucocorticoid and mineralocorticoid receptors, which may in turn inhibit synaptoplastic mechanisms associated with cognitive function in patients with depression [25]–[27],[59]. In the coronal hippocampal slice, tetanic simulation of the SC projection to CA1 pyramidal cells can induce LTP [9]. Tree shrews also showed robust LTP in this glutamatergic pathway, while social defeat stress led to LTP failure, providing a possible explanation for the cognitive deficits associated with social defeat [60],[61]. After 4 weeks of clomipramine treatment and 1 week of recovery, subordinate tree shrews still displayed impaired LTP induction and clomipramine did not rescue this deficiency. Tricyclic antidepressant can actually worsen memory dysfunction in depressed patients [62], again indicating that this drug may normalize some neurobiological processes, such as those associated with pleasure seeking, but not others.

### Side-Effects Of Clomipramine

While TCA has proven beneficial for many depressed patients over the past decades, there are serious side effects that may be intolerable or dangerous for some patients [63]. Significant weight gain or loss is a common depressive symptom [41]. Our present study showed that chronic social defeat could cause significant weight loss in stressed tree shrews. Previous clinical studies show that when administered long term, TCAs typically disrupt central appetite control centers dependent on cholinergic and histaminergic neurons [64], and changes in weight are a major reason for non-compliance or termination of therapy. In this study, clomipramine exacerbated weight loss in subordinate tree shrews, a response warranting further study to elucidate the neurobiological mechanisms mediating the effects of TCAs and other antidepressants on weight regulation.

In conclusion, subordinate tree shrews having experienced chronic social defeat exhibit many symptoms and behaviors similar to those observed in depressed patients, including weight loss, anhedonia, dysfunction of the HPA axis, fatigue, and agitated depression. In addition, these animals demonstrate impaired hippocampal LTP, a feature shared by rodent models of depression. The parallel effects of clomipramine on human and tree shrew responses are suggestive of this model’s robust predictive, face, and construct validity for investigating the etiology and pathophysiology of major depression.
